# P-1631. Physician Perceptions and Decision-Making Factors in Prescribing COVID-19 Antivirals: Qualitative Research Findings

**DOI:** 10.1093/ofid/ofaf695.1807

**Published:** 2026-01-11

**Authors:** Maria Fernandez, Domenick Francis, Iqra Naz Arham, Joshua R Coulter, Brett Hauber, Shoaib Khan, Lewis Kopenhafer, William You, Kathleen Beusterien, Martine C Maculaitis, Soohyun Hwang, Michael J DiGiovanna, Mary M Moran, Ruth Mokgokong

**Affiliations:** Pfizer, Chapel Hill, NC; Pfizer, Chapel Hill, NC; Pfizer, Chapel Hill, NC; Pfizer, Chapel Hill, NC; Pfizer, Inc., New York, New York; Pfizer, Chapel Hill, NC; Oracle America, Inc., Los Angeles, Virginia; Oracle Life Sciences, Austin, Texas; Oracle Life Sciences, Austin, Texas; Oracle Life Sciences, Austin, Texas; Oracle Life Sciences, Austin, Texas; DiGiovanna Family Care Center A Division of NY Health, Smithtown, New York; Pfizer Inc., Collegeville, Pennsylvania; Pfizer, Chapel Hill, NC

## Abstract

**Background:**

Despite the availability of effective antivirals, patients with COVID-19 at high risk of progression to severe COVID-19 remain undertreated, leading to avoidable patient- and system-level burden. We conducted a qualitative concept elicitation study to identify key drivers and barriers to prescribing antivirals, informing a future quantitative preference survey on factors impacting COVID-19 antiviral utilization.

**Table 1.**
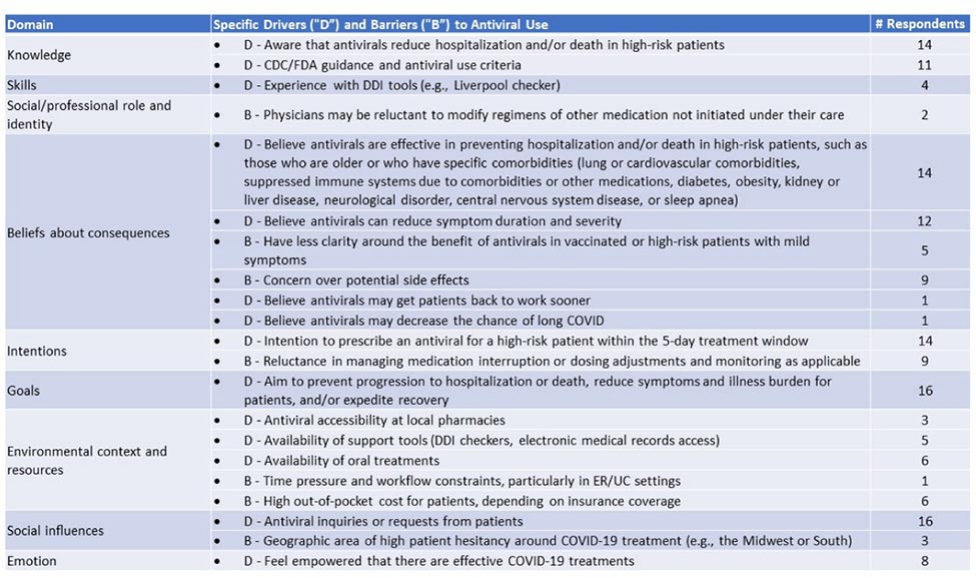
COVID-19 Antiviral prescribing drivers and barriers by relevant Theoretical Domains Framework domain

**Methods:**

Sixteen US physicians [6 primary care (PC), 5 urgent care (UC), and 5 emergency medicine (ER)] participated in individual 45-minute semi-structured virtual interviews with a moderator, guided by the Theoretical Domains Framework (TDF), a 14-domain framework for identifying determinants of behavioral change, between November 2024-January 2025. The interviews were transcribed and thematically analyzed using MAXQDA^®^ qualitative analysis software.

**Results:**

Concept saturation was attained by the 16th interview. Key drivers of antiviral prescribing included reducing hospitalization and/or death, along with symptom reduction for high-risk patients (older adults and those with comorbidities, such as cardiovascular disease, pulmonary conditions, immunosuppression, obesity, and diabetes) were key concepts identified. Oral antivirals were preferred over IV in an outpatient setting. Barriers included uncertainty about the benefits of antivirals in vaccinated patients or high-risk individuals with mild symptoms. Reluctance to modify medication regimens because of potential drug-drug interactions (DDIs) was also cited as a deterrent, particularly among ER and UC physicians, who face time constraints and complex treatment considerations. All physicians reported that patient preferences play a substantial role in antiviral prescribing decisions. Drivers, barriers, and the number of respondents who mentioned them within each TDF domain are summarized in Table 1.

**Conclusion:**

Physicians view antivirals as an effective tool for treating patients with COVID-19 at high risk for progression to severe COVID-19, yet this study identified factors that may deter prescribing. Further research is needed to quantify the influence of specific drivers and barriers to prescribing antivirals for COVID-19.

**Disclosures:**

Maria Fernandez, PhD, MBA, Pfizer Inc.: Employee and may hold stocks Domenick Francis, PharmD, Pfizer: Stocks/Bonds (Public Company) Iqra Naz Arham, PharmD, RPH, Pfizer: Stocks/Bonds (Public Company) Joshua R. Coulter, MA, Pfizer: Stocks/Bonds (Private Company) Brett Hauber, PhD, Pfizer Inc: Stocks/Bonds (Private Company) Shoaib Khan, MD, Pfizer: Stocks/Bonds (Public Company) Lewis Kopenhafer, BA, Oracle America, Inc: Employee William You, MSPH, Pfizer Inc.: Advisor/Consultant Kathleen Beusterien, MPH, Oracle America, Inc.: Employee Martine C. Maculaitis, PhD, MA, Oracle Life Sciences: Employee of Oracle Life Sciences, which received funding from Pfizer to conduct the study. Soohyun Hwang, PhD, MPH, Pfizer: Employed by Oracle Life Sciences, which received funding from Pfizer to conduct the study. Mary M. Moran, MD, Pfizer Inc.: Employee of Pfizer Inc. and may hold stock or stock options Ruth Mokgokong, PhD, Pfizer: Employee|Pfizer: Stocks/Bonds (Private Company)

